# Breast cancer brain metastasis: from etiology to state-of-the-art modeling

**DOI:** 10.1186/s13036-023-00352-w

**Published:** 2023-06-29

**Authors:** Mohammad Kamalabadi Farahani, Maliheh Gharibshahian, Alireza Rezvani, Ahmad Vaez

**Affiliations:** 1 Department of Tissue Engineering, School of Medicine, Shahroud University of Medical Sciences, Shahroud, Iran; 2 Student Research Committee, School of Medicine, Shahroud University of Medical Sciences, Shahroud, Iran; 3 Hematology Research Center, Shiraz University of Medical Sciences, Shiraz, Iran; 4 Department of Tissue Engineering and Applied Cell Sciences, School of Advanced Medical Sciences and Technologies, Shiraz University of Medical Sciences, Shiraz, Iran

**Keywords:** Brain metastasis, Breast cancer, Tissue engineering, Scaffold, Cancer modeling, Cancer cell lines, Animal models

## Abstract

Currently, breast carcinoma is the most common form of malignancy and the main cause of cancer mortality in women worldwide. The metastasis of cancer cells from the primary tumor site to other organs in the body, notably the lungs, bones, brain, and liver, is what causes breast cancer to ultimately be fatal. Brain metastases occur in as many as 30% of patients with advanced breast cancer, and the 1-year survival rate of these patients is around 20%. Many researchers have focused on brain metastasis, but due to its complexities, many aspects of this process are still relatively unclear. To develop and test novel therapies for this fatal condition, pre-clinical models are required that can mimic the biological processes involved in breast cancer brain metastasis (BCBM). The application of many breakthroughs in the area of tissue engineering has resulted in the development of scaffold or matrix-based culture methods that more accurately imitate the original extracellular matrix (ECM) of metastatic tumors. Furthermore, specific cell lines are now being used to create three-dimensional (3D) cultures that can be used to model metastasis. These 3D cultures satisfy the requirement for in vitro methodologies that allow for a more accurate investigation of the molecular pathways as well as a more in-depth examination of the effects of the medication being tested. In this review, we talk about the latest advances in modeling BCBM using cell lines, animals, and tissue engineering methods.

## Introduction

Breast carcinoma is the most common type of cancer and the main cause of cancer mortality in women [1–3]. Exposure to estrogen may have a role in the development of DNA damage and genetic alterations, both of which are necessary for the progression of breast cancer. Sometimes, cancer-causing DNA mutations or genes like BRCA1 and BRCA2 may be passed down from generation to generation. As a result, an increased likelihood of developing breast cancer is associated with having a history in one’s family of either ovarian or breast cancer. In a healthy person, the immune system will destroy cells that have abnormal DNA or are growing in an abnormal manner. When a patient has breast cancer, this strategy fails, allowing the tumor to develop and spread [3, 4]. Over the last 10 to 15 years, therapeutic approaches have changed to take into consideration the heterogeneity of the disease, with a focus being put on more biologically-directed treatments in order to minimize the deleterious effects of therapies [5]. Some characteristics, such as the influence of metastatic trends or the effect of the local tumor burden, are shared and have an effect on treatment, regardless of the fact that the underlying molecular heterogeneity is the guiding premise of present therapies. Breast cancer that is detected at an early stage is often treatable [6]. As a consequence of developments in multimodal therapy, the percentage of patients who will be cured after receiving treatment has climbed to somewhere between 70—80%. Nevertheless, metastatic breast cancer is a disease that can be treated, and the primary objectives of treatment are to increase the length of time a patient may live and to manage symptoms while minimizing treatment-related side effects in order to preserve or enhance the quality of life [6, 7]. New treatment strategies for breast cancer employ targeted therapies in combination with apoptotic ligands and chemotherapy. However, the recurrence and metastasis of breast cancer due to chemoresistance are major problems [8]. In breast cancer, metastasis and chemo-resistance are the most common causes of treatment failure. Therefore, elucidating the underlying mechanisms is crucial for developing new therapeutic strategies [9, 10].

**Fig. 1:**
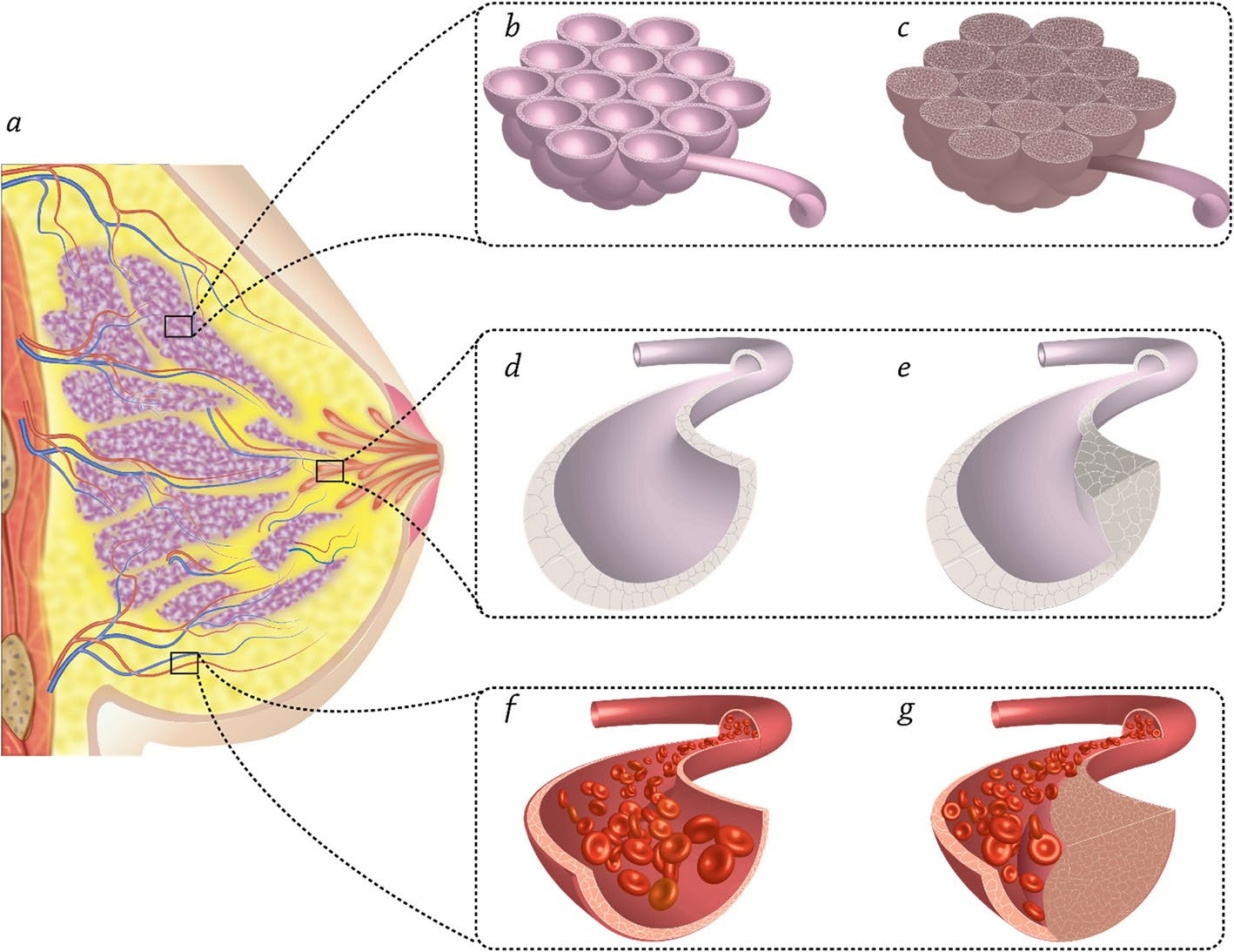
Schematic illustration of: **a** anatomical structure of the breast, **b** normal breast gland (lobule), **c** lobular carcinoma, **d** normal milk duct, **e** ductal carcinoma, **f** normal artery, **g** angiosarcoma

On a histological level, however, breast cancer may be broken down into two main subtypes: in-situ carcinoma and invasive carcinoma. The in-situ subtype is less common than the invasive subtype, which accounts for the majority of breast cancer cases. More than 80% of the invasive breast cancers are invasive ductal carcinomas (IDCs), and the remainder are invasive lobular carcinomas (ILCs) (Fig. 1) [11]. Both ILC and IDC have specific organ preferences when it comes to the spread of metastatic disease. ILC has three times more metastases in the peritoneum, gastrointestinal tract, and ovaries than IDC does, which tends to metastasize to the lungs, distant lymph nodes, and central nervous system (CNS) [12]. However, there are other subtypes of breast cancer like inflammatory breast cancer (cancer cells obstruct lymph capillaries in the skin, which makes the breast seem "inflamed"), angiosarcoma (endothelial cells of the blood or lymph vessels are the first to get affected by angiosarcoma), Paget disease of the breast (it begins in the milk ducts of the breast, then migrates to the skin of the nipple, and finally reaches the areola), and Phyllodes tumors (the majority are absolutely benign and originate in the breast’s stroma (connective tissue)) [13, 14].

Nevertheless, research focusing on tumor cell biology has demonstrated that histological abnormalities are not adequate prognostic indicators for metastasis risk if applied alone, without biological markers. Biological indicators differentiate breast tumors into molecular subgroups. These indicators are assessed by immunohistochemical staining (IHC) or microarray-based gene expression. Epidermal growth factor receptor (EGFR), human EGFR2 (HER2), cytokeratin 5/6 (CK5/6), and the cell proliferation marker Ki67 are some examples of hormone receptors (HRs) [4]. Other examples are progesterone receptors (PR) and estrogen receptors (ER). On the basis of these indicators, the various molecular subtypes of breast cancer are categorized as follows: luminal A (ER^+^ and/or PR^+^, HER2^−^, and Ki67 ^low^), luminal B (ER^+^ and/or PR^+^, HER2^−^, and Ki67 ^high^), luminal-HER2 (ER^+^ and/or PR^+^ and HER2^+^), HER2-enriched (ER^−^, PR^−^, HER2^+^), basal-like (ER^−^, PR^−^, HER2^−^, and EFGR^+^ or CK5/6^+^), and triple-negative (TN) phenotype (ER^−^, PR^−^, HER2^−^). TN has a high prevalence of p53, a tumor suppressor gene, mutations and 80% of them display basal-like characteristics, however, they are labeled TN-non basal [15].

## Breast Cancer Brain Metastasis (BCBM) mechanisms

Metastases account for 90% of human cancer deaths. In cancer treatment, metastasis and resistance to chemotherapy are linked phenomena [16]. Metastasis is a major cause of fatality, especially in breast cancer. Brain, axillary lymph nodes, bone, lung, and liver are the main sites of metastasis [17].

Breast cancer cells (BCCs) invade surrounding tissue and vasculature, migrate through the circulatory system, then colonize and multiply inside the brain parenchyma in order to develop brain metastases [18]. The breast cancer tumor cells, unlike other cancer cells, require more time to gain the capacity to break through the blood–brain barrier (BBB) and colonize the brain. Brain metastasis of breast cancer occurs in two stages: the tumor cells’ passage from the BBB and their progress inside the brain. Opening the tight junctions between tumor cells is known as the first step of their migration [19]. According to the studies, extravasation of tumor cells is affected by endothelial cells’ activity, and damage to the vascular walls does not play a role in this. Tumor cells are trapped in the endothelium, connect to the subendothelial matrix (as a result of intercellular interaction), and start the colonization process by modifying the surrounding microenvironment [19, 20]. Inflammatory cytokines, cell surface receptors, and adhesion molecules (such as integrin and selectin) promote the adhesion of tumor cells to the endothelium. Only a small population of CD44 + /CD24 − BCCs with an invasive phenotype and high expression of pro-invasive genes (such as IL-8, IL-6, and urokinase plasminogen activator) participate in this metastatic process [21, 22].

Stromal urokinase plasminogen activator and matrix metalloproteinase-2 (MMP-2), as inactive components, allow neoplastic cells to pass through the basement membrane. Urokinase plasminogen activator causes the conversion of plasminogen to plasmin, and in the next step, plasmin is suppressed by neuroserpin and serpin B2, and tumor cells pass through the BBB [23].

After crossing the BBB barrier, cancer cells interact with astrocytes (vital cells in maintaining BBB and brain homeostasis). Tumor cells use gap junctions of astrocytes and transfer the cyclic guanosine monophosphate adenosine monophosphate (cGMP) to them, resulting in the production of inflammatory cytokines (such as interferon alpha (INFα) and tumor necrosis factor-alpha (TNF-α)), and therefore signal transducer and activator of transcription 1 (STAT1) and activates nuclear factor kappa-light-chain enhancer of activated B cells (NF-κB) pathways is activated in metastatic cells and causes tumor growth and chemoresistance [24]. The direct contact of tumor cells and astrocytes also activates the Akt/mitogen-activated protein kinase (MAPK) pathway and causes the activation of anti-apoptotic genes in tumor cells [25].

Tumor cells, by secreting IL-1β, activate Notch in astrocytes and stimulate the Notch pathway. On the other hand, MMPs released by tumor cells also contribute to the development and growth of tumor cells by destroying collagen. And during a series of complex signaling pathways, tumor cells are finally implanted in the brain [26].

Even if the BBB is breached as a result of tumor invasion, many of the treatments that are now in use are unable to pass across this barrier, which contributes to the selective pressure that may make the brain a preferable location for metastasis [27]. Tumor cells in different steps of this process have distinctive and appropriative properties that will help them during this process [28]. Identification of these specific features could be useful in the designing of new therapies.

Several studies have focused on the molecular level of the metastasis process [29, 30]. In the metastasis process, metastatic tumor cells develop chemoresistance and radioresistance. Molecular biomarkers such as genetic changes, aberrant gene expression, and deletion or change of specific gene expression can help identify this metastasis to be proposed as an optional treatment for patients [31]. Examining brain metastasis tissues (resulting from biopsy) has shown the presence of unique markers in more than half of brain metastases, which were not present in primary tumor tissue (Table 1). Although many different molecular processes can lead to therapy resistance, and we don’t fully understand all of them yet [32], Some of the molecular dysregulation linked to BM are discussed here.

**Table 1:** Molecular biomarker of brain metastasis

Biomarkers	Main role
Change of ER/PR/HER2 expression	Hormone/ tyrosine kinase receptor
Phosphatase and tensin homolog (PTEN) deletion	regulating the phosphatidylinositol 3-kinase (PI3K)/AKT/mTOR pathway
Mutation in the cyclin-dependent kinases (CDK)	DNA replication and facilitates G1 to S phase transition
Downregulation of retinoblastoma protein 1 (RB1)	Tumor suppressor and G1 checkpoint

ER and PR are the main biomarkers in brain metastasis of breast cancer, and mutation and change in their expression can indicate brain metastasis. HER2 is also a tyrosine kinase receptor which is generally used with hormone receptors to identify breast cancer and its metastases. The ER- and PR-negative, the HER2-positive, and the negativity of all three parameters (TN) indicate an increase in the potential of brain metastasis. In other words, in brain metastases, the progesterone, estrogen, and ERBB2 gene expression is decreased, decreased, and increased, respectively [33]. These biomarkers’ expression is different in more than 22.8% of patients with primary breast cancer and brain metastasis. In addition, they are different in 63.6% of patients with brain metastasis and extracranial metastasis [34, 35]. Therefore, identifying them and reversing the aforementioned process is one of the main therapeutic goals of BCBM. For example, drugs with anti-HER2 effects (such as trastuzumab and lapatinib) can be valuable in brain metastasis treatment [36].

Some cancer stem cell (CSC) markers, including nestin, CD44 [37], and CD133 [38], have been linked to brain metastasis cells. In comparisons of primary breast cancers with metastases, a relatively high frequency of hypermethylated genes is reported in metastases to the bone, brain, and lung. For example, brain metastases are more likely to have hypermethylation of cyclin D2, retinoic acid receptor-, and hin-1 [18, 39]. Patients with HER2-positive and TN metastatic breast cancer are at a greater risk of developing brain metastases, with up to 50% of these patients developing brain metastases over time [40]. Patients with TN and HER2-positive tumors were shown to have shorter median time intervals between the main diagnosis and the development of brain metastases, while patients with ER-positive tumors had longer median time intervals [41, 42].

In addition to HER2, HER3 overexpression is also connected with brain metastases in breast cancers. The major ligand of HER3/HER2 heterodimers, heregulin (HRG), is abundantly expressed in the human brain and is able to promote the transendothelial migration of HER2/HER3-positive BCCs over a tight barrier of brain microvascular endothelial tissue [43]. Finally, MMP-9 has been identified as one of the elements partly mediating this process. Notably, in BCCs, HRG-induced MMP-1 and MMP-9 production is regulated via a HER3-dependent pathway, and cells with a greater amount of HER2 are more aggressive than those with a lower HER2 expression [44]. In a xenograft model, a possible profile of brain metastasis marker HER2 + /EGFR + /Heparanase (HPSE) + /Notch1 + in Epithelial Cell Adhesion Molecule (EpCAM)-circulating tumor cells (CTCs) was found to be highly invasive and able to spread to the brain and lungs [45].

As another marker of brain metastasis, we can mention changes related to chromosome 10 PTEN deletion in these patients. PTEN plays a role in regulating the PI3K/AKT/mTOR pathway, and in a patient with brain metastasis, the activation of this pathway increases the proliferation of tumor cells [46]. In addition, the reduction of PTEN expression in patients with brain metastasis makes cancer cells more sensitive to the inhibition of polyadenosine diphosphate ribose polymerase. PTEN expression in patients with brain metastases is significantly decreased compared to breast cancer patients and extracranial metastases patients. Therefore, effective treatments can be selected from agents that antagonize the PI3K pathway and inhibit polyadenosine diphosphate ribose polymerase (such as veliparib) [47].

CDK is a serine/threonine protein kinase involved in the G1 checkpoint regulation. During the G1 phase, CDK promotes DNA replication and facilitates G1 to S phase transition. Mutation in the CDK pathway is another parameter of brain metastasis [48, 49].

Homozygous RB1 is a tumor suppressor and G1 checkpoint that prevents rapid and uncontrolled cell division. The expression of this protein is generally downregulation in brain metastases and increased the invasive potential of tumor cells [50].

BCBM patients indicate a catastrophic occurrence that portends a bad prognosis regardless of the therapy that is undertaken. In particular, brain metastases are a substantial contributor to morbidity since they are linked with growing neurologic impairments that lead to a worse quality of life in the patient. Because of the development of more effective systemic treatments, brain metastases are becoming an increasingly common clinical issue. Patients BCBM have limited treatment choices, which include surgical resection, whole-brain radiation therapy, stereotactic radiosurgery, chemotherapy, and targeted therapy. Other treatment alternatives are unavailable [27].

## BCBM models

The brain microenvironment represents a distinct niche. Tissue-resident cell types such as neurons, oligodendrocytes, astrocytes, and microglia, as well as their particular metabolic and ECM features, may be encountered by metastatic BCCs. Moreover, the BBB protects the brain by acting as a natural filter for many molecules that are carried via the systemic circulation, such as anticancer drugs. In fact, the primary role of BBB is to maintain the homeostasis of the brain for proper neuronal functions [51].

Even though the barrier changes into a new entity known as the blood-tumor barrier (BTB), it nevertheless maintains the capacity for selective permeability. The convolution of the brain microenvironment is further compounded by the genetic and phenotypic heterogeneity of the brain metastatic cancer cells themselves. This is due to the fact that cancer cells may originate from a variety of source sites before settling in the brain. Therefore, the utilization of preclinical models that authentically reproduce the intricacy of this multiple processes is very necessary if one is interested in deciphering the genesis of CNS metastases and locating fresh treatment alternatives. Still, no model can solve all of the unanswered questions [52, 53].

Consequently, the factual issue that the researcher is trying to answer determines which model is the most appropriate to use. So, in order to evaluate possible new medicines or biomarker methods, we need models that accurately show the variety and complexity of medical problems as well as the clinical condition [54].

### Application of cell lines and animal models for BCBM research

#### Cell lines

It is vital to employ appropriate models that, to the greatest extent feasible, accurately recreate the course of the patient so that a molecular understanding of the genesis of CNS metastases may be achieved, as well as so that preventative measures and therapies can be developed. Thus, the intricacies of brain metastases necessitate that several models should be explored. In this regard, several cell lines have been developed for preclinical BCBM research [55]. Brain metastatic cell lines (BMCLs) have been generated from human or mouse parental primary tumors. Furthermore, spontaneous metastatic cells from genetically engineered mouse or human models or orthotopic injections are another source of BMCL [56]. Using data relating to experimental models of brain metastases obtained from 19 independent laboratories, the BMCLs can be categorized into 60 cell lines, obtained from patients (32 cell lines), mice (27 cell lines) or rats (1 cell line), and depict the three main sources of brain metastasis, including breast cancer (38 cell lines), lung cancer (8 cell lines) and melanoma (14 cell lines) [56].

BMCLs from breast cancer exhibit 3 molecular subtypes. BMCLs have all of the main somatic mutations reported in humans, including those impacting breast cancer gene 1 (BRCA1), phosphatase and PTEN, HER2, CDK, EGFR and p53, and others which are less frequent like BRAF (serine/threonine-protein kinase B-raf), MYC (proto-oncogene, BHLH transcription factor), KRAS (proto-oncogene, GTPase), RB1 and SMAD4 (SMAD family member 4). Expression of HER2 and absence of expression of ER are substantial risk factors for the development of brain metastases in breast cancer patients. Similarly, the bulk of the cell lines is from TN and HER2^+^ subtypes [46, 57–59].

Accordingly, in an effort to develop xenogeneic models that selectively form brain metastases, cell populations have been selected that have a tendency to establish BCBM, such as mucin (MUC1) secreting MA11 cell line derivatives, which after intracardiac injections in BALB/c nu/nu mice preferentially form BCBM in 87% of animals [60]. The extensive depiction of breast cancer models in the BMCLs includes cell lines that grow rapidly in the brain. These models will provide the brain metastatic research community with tools to better portray the heterogeneity of brain metastases [57]. By clonally selecting cell populations from parental immortalized BCCs lines with a predisposition to developing brain metastases, it is possible to boost the effectiveness of BCBM production. A parental ER/PR/HER2-, or TN, MDA-MB-231 cell line was administered intracardially into nude mice to create the brain-seeking clone [61]. Three to four weeks later, cells from brain metastases were grown in vitro and re-inoculated into the animals. The brain-seeking MDA-MB-231BR (231BR) cell line was produced after six iterations of this method, with 100% rate of brain metastases and no metastases to other organs [62]. Three rounds of selection and intracarotid administrations in mice were used to create further subclones of the 231BR cell line, yielding the BR1, BR2, and BR3 sublines. These sublines were distinct from the initial 231BR cells in that they produced higher amounts of VEGF-A, which has been demonstrated to be essential for the growth of BCBM [63]. In fact, relative to the 231BR cells, they caused mice to live shorter lives and to acquire more brain metastases [64]. Similar methods of intracardiac injections and clonal selection via a second round of in vitro and in vivo culture were used to produce the MDA-MB-231-BrM2 subline, which resulted in metastases in the cerebellum, brainstem, cerebrum, and leptomeninges [65]. A CN34-BrM2 clone that metastasized to the same sites in the mouse brain after intracardiac or mammary fat pad injections was published using an identical strategy but a different TNBC cell line, CN34 [65].

**Fig. 2:**
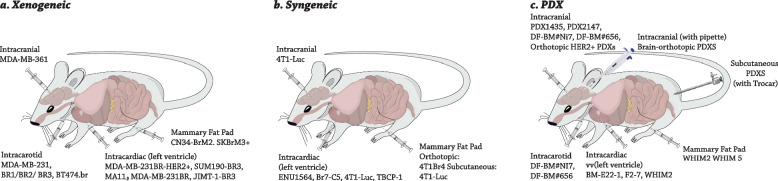
Techniques for implanting cancer cells in an in vivo model in order to produce BCBM models. Xenogeneic models (**a**), syngeneic models (**b**), and patient-derived xenograft models (**c**) are some of the ways that diseases have been introduced

Also, a cell line containing MDA-MB-231BR-HER2^+^ (231BR-HER2^+^) has the potential to grow BCBM more rapidly and to produce more large metastatic tumors in BALB/c nude mice than the 231BR cell line [66]. There have also been descriptions of other HER2 + brain-seeking sublines that are based on the JIMT-1, SUM190, and BT474 lines that can potently develop the BCBM (Table 2) (Fig. 2a) [67, 68].

**Table 2:** Cell lines for BCBM preclinical models

Models	Cell Type	Subtype	**Animal model**	**References**
**Xenogeneic**	MA11	TN	BALB/C nu/nu nude mice	[69]
	MDA-MB-231BR	TN	Nude mice	[70–72]
	MDA-MB-231BR1, -BR2, -BR3	TN	Athymic NCr-nu/nu mice	[73]
	MDA-MB- 231-BrM2	TN	Athymic nude mice	[74]
	MDA-MB-231BR-HER2 +	ER-/PR-/HER2 +	BALB/c nude mice	[75, 76]
	MDA-MB-361	ER + /PR + /HER2 +	Nude mice	[77]
	MDA-MB-468	TN	Nude mice	[78]
	CN34-BrM2	TN	Athymic nude mice	[79]
	JIMT-1-BR3	HER2 +	NRC nu/nu mice	[73]
	SUM190-BR3	HER2 +	Athymic NIH nu/nu mice	[80]
	BT474.br/Br.2/Br.3	ER + /PR + /HER2 +	Swiss nude mice	[81, 82]
	SKBrM3 +	ER-/PR-/HER2 +	Athymic nude mice	[83]
**Syngeneic**	4T1BM	TN	Syngeneic BALB/c mice	[84]
	4T1Br4	TN	Syngeneic BALB/c mice	[85, 86]
	4T1-Luc	TN	Syngeneic BALB/c mice	[87]
	Br7-C5	Unspecified	Berlin–Druckrey IV rat	[88]
	TBCP-1	ER-/PR-/HER2 +	Syngeneic BALB/C mice	[89]
**PDX**	F2-7	TN	NSG mice	[90]
	Brain-orthotopic PDXs	TN and ER + varied	NSG mice	[91]
	BM-E22-1	TN	NSG mice	[90]
	DF-BM#Ni7, DF-BM#656	ER + HER2 + (DF-BM#Ni7), TNBC (DF-BM#656)	NOD/SCID mice	[92]
	WHIM 2/WHIM5	TN	NOD/SCID mice	[93]
	PDX1435/PDX2147	TN	NOD/SCID mice	[70]
	Orthotopic HER2 + PDXs	HER2 + , ER/PR status varied	NOD/SCID mice	[94]
	Subcoutaneos PDXs	Unspecified	SCID BALB/c mice	[95]

Research on syngeneic models has been conducted as a method of compensating for the deficiencies caused by the lack of immune system components in xenogeneic models of BCBM. The discovery of new immunotherapies and the subsequent implementation of these therapies into clinical practice for the treatment of metastatic breast cancer highlight the significant value of syngeneic models [96]. In this sense, Br7-C5, a brain-seeking clone that was derived from the ENU1564 rat mammary cancer cell line, is responsible for nonspecific metastases to the brain [97]. 4T1Br4, a brain metastatic subline of 4T1 cells, had a greater rate of metastasis to the brain (20%) than the original 4T1 (7%) [98]. A model that is based on 4T1 cells and uses either intracranial or intracardiac injection of luciferase-transduced 4T1 cells into mice has also been published. Compared to subcutaneous injection (Fig. 2b), this model led to more BCBM (25%) than subcutaneous injection [68, 99]. Despite all the benefits, these cell lines can’t fully reproduce the clinical and biological differences between human tumors (Table 2) [100, 101].

Patient tissue-derived models, such as patient-derived xenografts (PDX), have been created in order to better reflect the variety of diseases as well as the therapeutic responses of individual patients. These platforms will provide the foundation for the subsequent generation of preclinical applied studies as well as personalized medicine [102]. In the BM-E22-1 TN breast cancer model, tumor tissue was generated by implanting cancer cells into the mammary fat pad of NSG mice. This model was used to study TN breast cancer. Following two generations, the tumors were dissociated into single cells and injected intracardially. Following 2–3 months after the injection, MRI scans revealed that half of the mice had developed macrometastases, while all of the animals had grown micrometastases. The WHIM2 and WHIM5 models were created using tissue obtained from a TN breast cancer primary tumor and brain metastases, respectively, collected from the same patient. These cells were then implanted into the mammary fat pads of NOD/SCID mice [103]. In a subsequent experiment, xenografts obtained from WHIM2 were xenotransplanted using intracardiac injections in order to produce BCBM. As mentioned, metastases formed in the brains of all of the mice in this model; however, the mice also developed metastases in other parts of their bodies, including the liver (50%), the lungs (33%), the ovaries (83%), and the adrenal glands (25%) (Table 2) (Fig. 2c) [68, 93].

#### Animal models

Animal models that do not reproduce patient conditions mislead the preclinical study results, which consequently lead to the failure of clinical trials that not only waste resources, but, even worse, expose patients to ineffective interventions [104].

While genetically engineered mouse models of breast cancer have significantly contributed to the identification of the roles of specific genes in tumor development, they have a low incidence of brain metastasis and do not fully reflect the disease in humans [104, 105]. Though brain metastases in mice can be generated by directly injecting cells into the blood circulation through the tail vein or into the heart. When cells are injected in this way, they spread through the body and end up in organs like the brain [106].

In the area of brain metastasis, mouse models have been the most extensively investigated. Various in vivo models, on the other hand, may supplement or even outperform mouse models in specific research issues [105]. The ENU1564 (rat model) and MDA-321br (human model), are two well-established brain-metastatic BCCLs for rat models. Due to the rat brain’s larger size compared to the mouse brain, these models have been shown to be especially useful for imaging approaches research. In the study of cancer neuroscience, rat brain metastasis models may also be beneficial [107, 108].

Regardless of the origin of cancer, brain metastasis research has also used non-rodent models. When combined with fluorescently tagged cancer cells, the optical transparency of the zebrafish makes it possible to quantitatively examine the spatio-temporal patterns of metastasis at a single-cell level. Zebrafish is suitable candidate organisms for high-throughput genetic screening for putative mediators of metastasis because they can easily reproduce and can be genetically manipulated [109–111]. Drosophila melanogaster is another non-rodent model organism that has been used to study brain metastases. Overexpression of oncogenic RasV12, inactivation of the cell polarity gene Dlg, and GFP in Drosophila eye discs led to tumor formation as well as invasion of surrounding brain tissue. Similar to zebrafish, Drosophila provides an appropriate platform for high-throughput genetic screening by simply crossing any RNAi fly line with the above-mentioned fly line [112, 113].

### BCBM tissue engineering models

Every year, hundreds of thousands of people lose their lives because of metastatic brain cancer. There are several reasons why it is critical to understand the biology and process of metastasis and how these factors impact metastasis, given that more than 90% of cancer fatalities are caused by this process [114, 115]. Metastasis can occur only when tumor cells have appropriate interactions with the target tissue microenvironment. The tumor microenvironment (TME) has a diverse population of tumor cells and immune cells, irregular vascularization, low nutrients, a gradient of growth factors, and hypoxia. This microenvironment could have an effect on how cancer cells grow and spread [114, 116].

On the other hand, only 10% of anti-cancer drugs can enter the market, while the cost of producing new drugs is about $ 2.7 billion. Most of these drugs fail in the clinical phases at a high cost and in a lot of time (up to two-thirds of the cost mentioned). This is because there are not enough accurate tumor models used in the lab phases. Therefore, in vitro tumor models that can mimic the biophysical and biochemical properties of TME are a reliable and cost-effective way to test drugs [117, 118].

A wide range of two-dimensional (2D) to three-dimensional (3D) models have been used for studying the invasiveness, metastasis, and drug screening of breast cancer [119]. Traditional 2D models are a convenient and inexpensive platform, but they cannot mimic the TME and cell–cell and cell-ECM interactions suitably. These models lack the complex 3D multicellular structure of the tumor in the body, and therefore their proliferation, differentiation, migration, and drug sensitivity are also different from typical tumors. In 2D culture, the drug reaches tumor cells easily and can kill them quickly. While hypoxia, low pH, and nutrient flow in the tumor can cause the expression of multidrug resistance proteins and thus increase the tumor’s resistance to chemotherapy. Also, tissue culture plastics are stiffer than brain tissue on the GPa scale, so they shouldn’t be used to study brain tissue [120–125].

Despite the abovementioned advantages, animal models cannot mimic the conditions and biology of the human body. Studying the behavior of human cancer cells in animal models requires animals with suppressed immune systems, which does not suggest the possible role of immune cells such as macrophages in tumor progression. In addition to these disadvantages, using them may be time-consuming and costly, and it also raises ethical concerns [126, 127]. On the other hand, 3D tissue engineering models, as a new generation of cancer models to study cancer biology and drug screening, can mimic tumor morphology, microenvironmental characteristics, multidrug resistance protein expression, and cell–cell and cell-ECM interactions [121, 128].

In this sense, spheroidal, organoid, hydrogels, scaffolds, bioreactor-based models, 3D printing and bioprinting models, cancer-on-a-chip models, and metastasis-on-a-chip models are examples of cancer and metastasis 3D modeling.

#### Spheroid models

A tumor spheroid consists of the aggregation of tumor cells in a 3D structure under non-adhesive culture conditions [129]. These self-assembled spheroids have morphology, cellular interactions, chemical resistance, and growth kinetics similar to tumors. The production of spheroids may be accomplished by a variety of approaches, including the hanging drop, suspension culture, liquid-overlay, and encapsulation procedures. However, there is not a lot of control over the size of the spheres or their homogeneity. It is possible to generate tumor spheroids by using a specific cell line or a combination of cell types [130–133].

Yuhas et al. evaluated the formation of spheroid BCCs isolated from different organs using the agar-based method. They isolated the MDA-361 cell line from human brain metastases. All cell lines derived from human solid tumors (including brain metastases) were able to form tumor spheroids. According to their results, MDA-361 has a faster growth rate in tumor spheroid than in a monolayer culture [134].

Ivascu et al. explored the adhesion molecules involved in rBM-driven versus spontaneous spheroid development in different populations of eight BCCs lines important for anticancer drug testing in preclinical studies. Spheroid production was inhibited in the presence of adhesion molecule functional blocking antibodies, as well as following siRNA-mediated downregulation of E- and N-cadherin proteins, and integrin 1 adhesion receptors. E-cadherin is involved in the spontaneous production of spheroids in MCF7, BT-474, T-47D, and MDA-MB-361 cells, but N-cadherin is involved in the tight packing of MDA-MB-435S cells. whereas the collagen I/integrin 1 association was predominantly responsible for the matrix protein-induced change in 3D cell aggregation into spheroids in MDA-MB-231 and SK-BR-3 cells, with no cadherin interaction. In MDA-MB-468 cells, a combination of homophilic E-cadherin and integrin/collagen I interaction formed spheroids [135].

It has been shown that SHH subgroup medulloblastoma, the most common malignant pediatric brain tumor, cell lines developed tight, highly reproducible 3D spheroids that could be maintained in vitro for a few weeks and formed pathological oxygen gradients when grown under the same stem cell enrichment conditions. In comparison to 2D models, 3D spheroid culture boosted resistance to standard-of-care chemotherapeutic treatments. Through dual-inhibitor experiments and continuous drug response monitoring, they demonstrated how this model can improve in vitro therapeutic screening methodologies. Then, using hyaluronan hydrogel matrices that were specific to the brain, they were able to build a metastatic cellular structure in the early stages of migration [136].

**Fig. 3:**
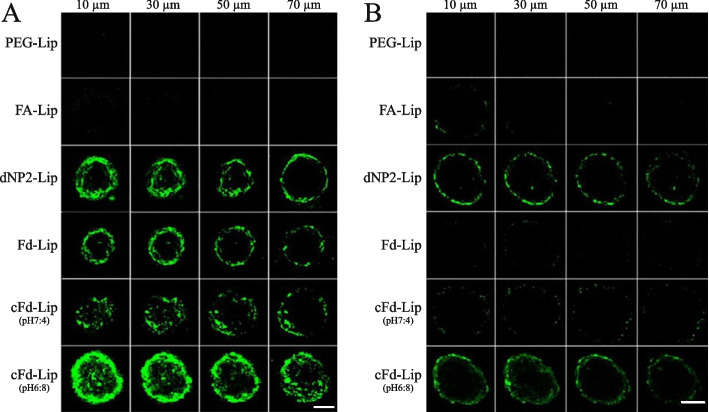
The capacity of CFPE-labeled liposomes to penetrate 4T1 (**A**) and 4T1&NIH 3T3 (**B**) tumor spheres. Scale bar, 100 μm. Printed with permission from ref [137]

According to reports, the high density of solid tumor cells and the intricacy of the TME often end up in poor medication dispersion, posing a formidable barrier to successful cancer therapy. The microenvironment of solid tumors was made up of cancer-associated fibroblasts (CAFs), which led to the creation of TME. Spheroids produced with tumor cells, on the other hand, cannot adequately imitate the TME. The existence of BBB and CAFs that modify the TME constitute significant hurdles in treating breast cancer and its brain metastases. Li et al. developed a technique for improved administration to orthotopic breast cancer and brain metastases employing a PTX-loaded liposome co-modified with acid-cleavable folic acid (FA) and BBB transmigrating cell-penetrating dNP2 peptide (cFd-Lip/PTX). cFd-Lip demonstrated enhanced TME localization and BBB transmigration when compared to single ligand or non-cleavable Fd-modified liposomes. Furthermore, when the acid-cleavable cFd-Lip/PTX reached the TME, it demonstrated sensitive cleavage of FA, thus minimized the hindrance function and increased the performance of both FA and dNP2 peptide. As a result, effective targeting of folate receptor (FR)-positive cancer cells and FR-negative CAFs was accomplished, resulting in increased anti-tumor action. This technique allows for cascade targeting of TME and BBB transmigration in orthotopic and metastatic cancer therapy (Fig. 3) [137].

For the purpose of studying mass dormancy in BMBC, a recent study created an in vitro hyaluronic acid (HA) hydrogel-based model. To replicate the brain ECM, HA hydrogels with a stiffness of 0.4 kPa were used. BT474Br3 or MDA-MB-231Br3 On top of HA hydrogels or in suspension, BMBC spheroids are created and cultivated for 7 days. By striking a balance between growing and dead cells, HA hydrogel generated a near mass dormant state in spheroids. These spheroids, on the other hand, grew in suspension cultures. In HA hydrogels, the ratio of %p-ERK to %p-p38 positive cells is substantially lower than in suspension cultures. It is also established that the hydrogel-induced bulk dormant condition is reversible [138].

#### Organoid models

Organoids could be used to incorporate microenvironmental elements into in vitro cell culture in diseases like metastasis brain cancer. This coculture preserves physiologically specialized cell–cell interactions that are not available in standard 2D cell culture techniques [139]. Organoids produced from patients also have the same phenotypic, genetic, and transcriptome heterogeneity as the original malignancies [140]. Investigators may be able to discover different routes of precision therapy if tumor complexity is preserved. Patient-derived organoids have recently been employed as an in vitro system that mimics the properties of patient-specific malignancies [141, 142].

Human tumor organoids may be created either directly from tumor sites or by genetically modifying organoids created from healthy tissues, like via the CRISPR technique. Organoids are created from tumor sites by growing cells that have been separated from the site in 3D ECM scaffolds in a specified medium containing adequate growth factors [143, 144]. Different kinds of common tumors have been used to make organoids that look and act like the original tumors [145].

Moreover, the production of chemokines by CAFs has been linked to increased angiogenesis and cancer cell migration. Chung et al. isolated and grew fibroblasts generated from healthy breast, primary, and brain metastatic tumors to study the involvement of CAFs in BCBM. In 3D organoid aggregates, the expression of numerous chemokines and growth factors have been studied using RNA-Seq, real-time quantitative qPCR, immunohistochemical staining, and ELISA testing. These results show that human brain metastasis CAFs attract BCCs through the chemokines CXCL12 and CXCL16 and that blocking the interactions between CXCR6-CXCL16/CXCR4-CXCL12 receptors and their ligands may be a good way to stop BCBM [146].

**Fig. 4:**
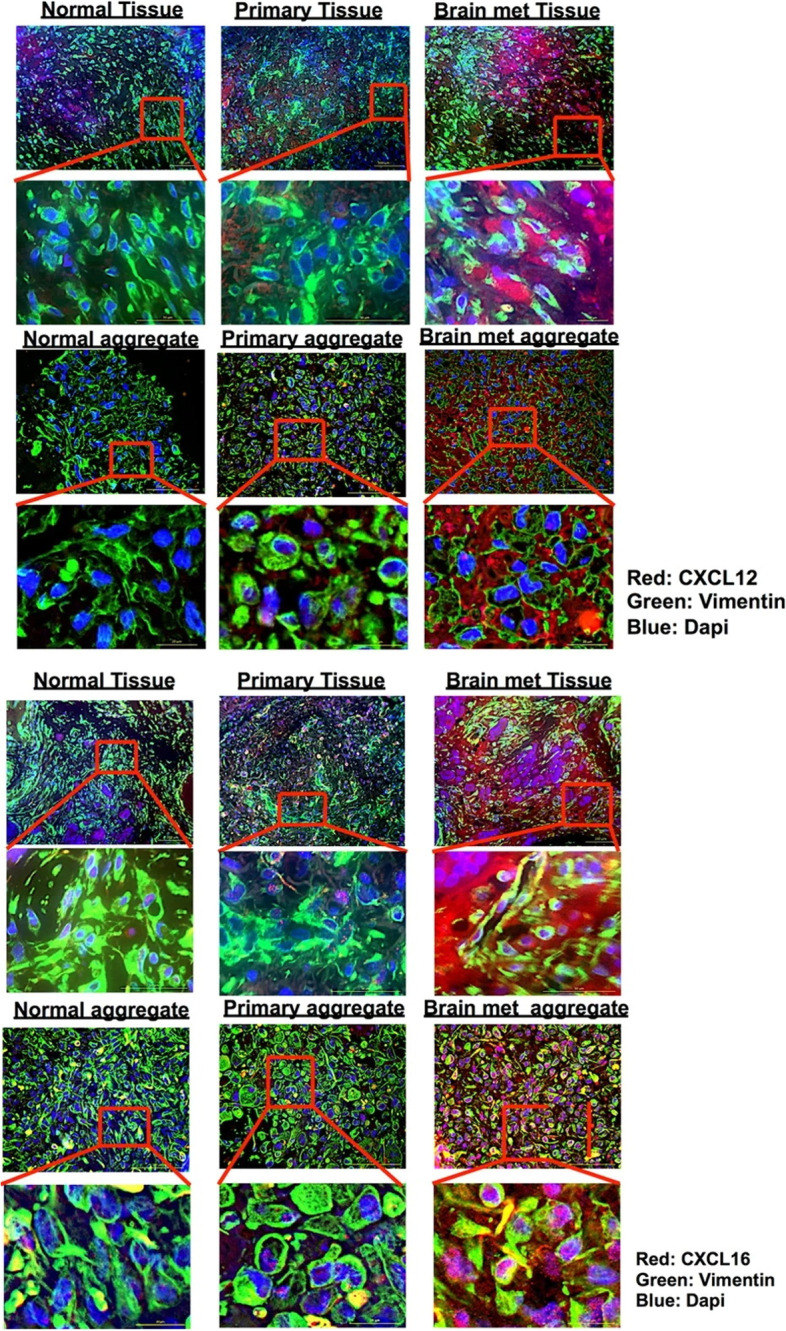
Immunofluorescence staining directed against the expression of CXCL12 and CXCL16 in patient tissue and patient-derived CAF aggregate Vimentin (green) and CXCL12/CXCL16 (red) expression may be seen in both patient tissues and patient-derived stromal aggregates in representative immuno-fluorescence images. Scale bar for zoomed images represent 50 µm. Reprinted in part with permission from ref [147]

CAF obtained from human BCBM were also shown to exhibit considerably greater amounts of the chemokines CXCL12 and CXCL16 when compared to fibroblasts derived from primary breast cancers or normal breast. This was discovered by Chung et al. via the use of RNA-Seq and protein analysis. They produced 3D organoids using patient-derived primary or brain metastatic cancer cells together with matching CAF in order to gain a deeper understanding of the interaction between cancer cells and CAF from each location. CAF aggregates created from primary tumors or normal breast stromal cells do not stimulate migration of cancer cells as efficiently as 3D CAF aggregates generated from brain metastases. These aggregates enhance migration of cancer cells. Treatment with a CXCR4 antagonist and/or a CXCL16 neutralizing antibody, either alone or in combination, strongly suppressed the migration of cancer cells to brain metastatic CAF aggregates. This was true whether the treatment was administered individually or in combination (Fig. 4) [147].

#### Hydrogels

**Fig. 5:**
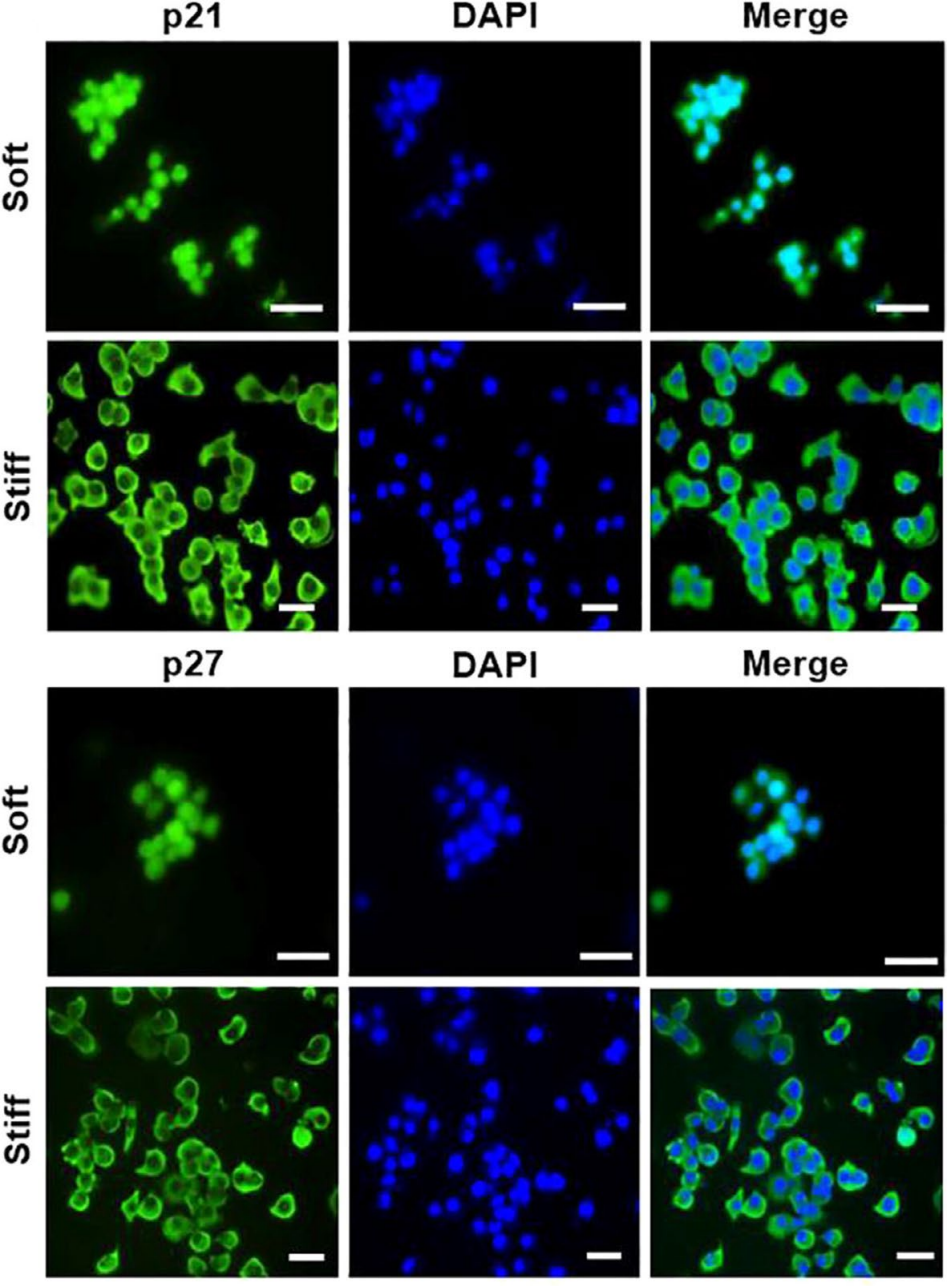
Immunofluorescence staining showed that p21 and p27 are found in the nucleus of MDA-MB-231Br cells when they are cultivated on soft (0.4 kPa) HA hydrogels, but they found to have cytoplasmic localization when they are grown on stiff (4.5 kPa) HA hydrogels. Representative fluorescence microscopy images of p21 (upper panel) and p27 (lower panel) staining at day 2 of MDA-MB-231Br cells cultivated on soft (0.4 kPa) and stiff (4.5 kPa) HA hydrogel, respectively. These pics were taken from MDA-MB-231Br cells that had been cultured for 2 days. Green: p21 or p27; Blue: DAPI (nuclei). Scale bar = 100 μm. Reprinted in part with permission from ref [149]

Drug delivery systems and smart hydrogels are two ways that use hydrogels as a primary tool in cancer treatment. Hydrogels’ rheological characteristics are also critical for optimizing the physical and mechanical behaviors of hydrogel systems. The study of cellular mechanotransduction and the behavior of delivery systems highly depends on these rheological and mechanical features. There are several factors that influence the rheological properties of hydrogels, such as the chemical composition, concentration of polymers and cross-linkers, the density of cross-linkers, and the degree of substitution. In this context, the biophysical signals offered by HA hydrogels were used in a study to replicate dormancy in BCBM cells using this hydrogel platform while classifying the normal brain and the stiffness related to metastatic brain malignancy [148]. It has been shown that MDA-MB-231Br and BT474Br3 BCBM cells show a dormant phenotype while cultivated on a soft (0.4 kPa) HA hydrogel compared to a stiff (4.5 kPa) HA hydrogel. Dormant MDA-MB-231Br cells were shown to have nuclear localization of the markers p21 and p27 (associated with dormancy), in contrast to the cytoplasmic localization of these markers in the proliferating population. Stiffness dormancy in MDA-MB-231Br cells was shown to be reversible and to be influenced by focal adhesion kinases and the initial cell seeding density. The inactive phenotype of MDA-MB-231Br cells was ultimately validated using RNA sequencing. Our knowledge of dormancy in BCBM might be improved by this platform, which could be used to test anti-metastatic drugs (Fig. 5) [149].

#### Scaffolds

Tissue engineering scaffolds are constructs that can be made of various biocompatible materials that mimic the natural ECM, allowing cells to attach, grow, and proliferate [125, 150]. It has been shown that tumor cells have a more aggressive phenotype and higher resistance to chemotherapy on scaffolds [151–153]. Structural, physical, chemical, topographic, and superficial signals of hydrogels can affect tumor cells’ function, gene expression, migration, and invasion. Hydrogels have relatively large interconnected pore sizes that allow cells to penetrate. They are attractive because of their high water content that are similar to brain tissue and also their adjustable chemical, physical, and mechanical properties [149, 154–156].

In this sense, Sualyneth Galarza et al. developed a 4-arm PEG-maleimide hydrogel to study BCBM by mimicking the biochemical and mechanical features of the brain. They did not see any significant differences in tissue stiffness between the tested species, but they could see modulus heterogeneity between the normal and metastatic tissues. By adjusting the weight percentage of the polymer, they adjusted the hydrogel mechanical properties to be the same as the natural tissue of the brain (Young’s modulus of 1.9 ± 1.7 kPa). They also added cell-binding site peptides to hydrogels to facilitate cell attachment, proliferation, and migration. Their results showed that this was a good way to study how tumor cells and healthy brain tissue work together [157].

Since there are so few CTCs, the broad adoption of CTC capture poses several hurdles, even though the technology seems attractive. Early-stage metastatic cells or foci may be identified early enough to allow for focused treatment modalities before distant organs have been compromised, which might result in longer distant metastasis-free prospects [158]. Implanting a scaffold that can attract metastatic cells is a creative project for early diagnosis. These cells, as well as soluble factors and some components of the ECM, are thought to form a niche favorable to tumor cell homing and colonization. According to these findings, metastasis is not a random process but is affected by the characteristics of the environment in which it occurs [159].

In this regard, the use of biomaterials and the production of scaffolds by them can be effective in trapping tumor cells. Poly(ε-caprolactone) (PCL) [159] and poly(lactide-co-glycolide acid) (PLGA) [160] scaffolds were developed to investigate the dynamic immunological reactions and biological processes associated with the scaffold-mediated attraction of metastatic BCC, as well as the effect of these scaffolds on the tumor microenvironment.

Moreover, implants for targeted drug administration, like drug-loaded nanoparticles [161–163], films [164, 165], fibers [166, 167], and gels [168], have also been created to increase chemotherapy effectiveness and minimize the likelihood of cell migration and invasion induced by remaining cancer cells and CTCs.

Also, many markers of cancer, including invasion and metastasis, have been found to be regulated by the ECM, which gives a structural framework to all tissues. The intricacy and heterogeneity of the tumor matrisome have been shown by the latest advancements in ECM proteomics [169]. Even after decades of research, it is still difficult to understand how the ECM influences cancer growth. Using recombinant proteins, cell attachment, migration, and invasion in individual ECM proteins have been examined in vitro. Adherence to ECM proteins as a scaffold is more predictive of 3D invasion than migration on the same 2D platform [170]. Using decellularized ECM structures from mammary gland tumors, WISHART et al. identified and studied ECM proteins involved in inducing breast cancer metastasis using live imaging. This procedure included isolating decellularized ECM structures and reseeding cells. When their method is combined with research on proteomics and mechanistic signaling, the effect of the ECM on the activity of tumor cells can be studied. ECM proteins that contribute to local invasion and how they work may also be studied [171].

#### Bioreactor-based models

Research into the effects of fluid flow-induced shear on cell migration is critical to understanding tumor exocytosis, as well as how cells adapt to dynamic distal migratory environments such as vascular and lymphatic systems. Cell density, rate of flow, cell receptor function, and geometry all influence malignant cell motility in the bioreactor system. In this context, MDA-MB-231 cells displayed varying behaviors to flows due to the environmental characteristics [172]. It has been shown that fluid shear in a 3D environment may improve the mobility of tumor cells. In this regard, Riehl et al. found that flow caused highly metastatic MDA-MB-231 cell migration along the flow direction with larger displacement, faster speed, and less stopping in a parallel plate flow chamber producing 15 dyne/cm2 shear stress. Whereas benign MCF-10A exhibited the lowest propensity for migration under shear and less metastatic MDA-MB-468 was less susceptible to flow [173].

#### 3D printing and bioprinting models

Personalized medicine can be achieved through the use of 3D printing, an additive manufacturing technology that enables the creation of 3D objects of practically any form. 3D printing is a simple and cost-effective method for developing controlled delivery systems that has significant features. In this sense, a pH-responsive, 3D printed matrix of PLGA, gelatin, and chitosan contains anti-cancer medications that are released in a regulated manner to minimize the risk of cancer spread and recurrence by local hemostasis and uptake of free cells. Aside from that, the implant may speed up the healing process, which can improve a patient’s prognosis if the incision is still open [174].

The absence of a vascular compartment, which plays an essential function in the process of supplying cells with nutrients and oxygen in the same way that it does when a tumor grows naturally, presents a significant obstacle to the development of 3D cancer models. The capability of the bioprinting method to incorporate cancer cells into a vascular network is a significant benefit that sets it apart from similar approaches [175]. For example, blood capillary architecture may be manufactured via the use of sacrificial bioprinting. In this process, microchannels are first constructed inside hydrogel foundation matrices through the careful elimination of bioprinted fugitive bionics. Following this step, endothelial cells are seeded into the inner surface of the previously stated microchannels to simulate the formation of natural blood capillaries. After this step, tumor micro-tissues may be placed in the hydrogel close to the bioprinted microvessels, which would then make it possible to monitor the neovascularization of the tumor [176].

The bioprinting method has high production efficiency, large-scale production capability, the ability to accurately mimic the characteristics of the TME such as stiffness, the ability to integrate vascular structures into the structure, and precise control over growth factors in the environment. Bio-printing is a valuable method in drug screening and the study of tumor biology [177–181]. Bioinks are made from various materials such as natural and synthetic polymers, hydrogels, microcarriers, and decellularized ECM [182, 183]. Bioink should be selected and designed based on the mechanical and physiological properties of the target tissue. Bioprinting methods include inkjet printing, micro-extrusion printing, laser-induced transfer printing, and stereolithography [184, 185]. Microfluidics and stereolithography bioprinting are two other methods of printing that have been used in the field of bioprinting [186]. In the first method, single and multilayered hollow tubular networks could be bioprinted in the first step, and then in the second step, they could be cellularized, which would ultimately result in the production of an extensive network of blood capillaries. This method was chosen because it was more straightforward [186, 187]. In the latter method, high-resolution capillary networks with the capacity to include new biomaterials may be generated to represent the hierarchical and chaotic tumor neovasculature. This method also has the advantage of being simpler [175, 188].

#### Cancer-on-a-chip models

Microfluidics is developing as a possible substitute in fundamental and applied biomedical science, with a broad variety of purposes. Oncology is primarily concerned with recognizing and using tumor-specific features, and microfluidic systems are well adapted to this task. Instead of using conventional 2D cell cultures or experimental animal studies, microfluidic systems allow researchers to better regulate the micromilieu of cells in a way that standard 2D cultures cannot [189]. Microfluidic systems make it possible to establish 3D cellular co-cultures that accurately simulate the micromilieu of in vivo tissues. These devices have the capability of simulating the development of breast cancer, as well as its growth and metastasis, such as its invasion, intravasation, and extravasation [190]. The 3D Cancer-on-a-Chip models are multi-channel microfluidic cell cultures made of glass, metal, and transparent polymers like polydimethylsiloxane (PDMS), poly(methyl methacrylate) (PMMA), polystyrene (PS), and polycarbonate (PC). These chips are made by various methods such as etching techniques, molding, 3D printing, and laser ablation. Simplicity, low cost, high speed and time-efficient, the need for fewer cells and animals, reproducibility, and the ability to closely mimic and control TME are some of the advantages of using these chips for cancer studies. These chips are used in drug screening, studying the influence of TME on metastasis, studying different stages of tumor development, and real-time monitoring. Cancer-on-a-chip models can also mimic the vascular and lymphatic structure within the tumor, co-culture and interactions of various tumor cells, the tumor vessel’s shear stress, the tumor stiffness, interstitial fluid pressure, and oxygen and chemical gradient inside a tumor [191–195]. In this case, Khademhosseini et al. designed the breast cancer-on-a-chip platform and studied the cardiotoxicity (one of the main side effects of chemotherapy) using induced pluripotent stem cells (iPSCs) -derived cardiac cells and cancer cells. Their results showed that this platform can be used to find and predict how chemotherapy will affect the heart [196].

Moreover, a microfluidic system was used to replicate the development and progression of breast cancer tumors. This was accomplished by reproducing the pathophysiologic vasculature that is present in patients. It was shown that low-perfusion physiology promotes the progression of tumors [197]. A similar device was also used to investigate the metabolism of cancer cells throughout the process of carcinogenesis. The results of this investigation showed that diverse responses to metabolic stress in aborning tumors reduce the effectiveness of anti-cancer therapies that address cancer metabolism [198]. In a generalized breast tumor analysis microfluidic system, breast cancer spheroids were also generated [199]. This was done for the purpose of early tumor physiological study and drug development.

#### Metastasis-on-a-chip models

The absence of strong technology for early screening of metastatic activities is a major impediment to the development of life-saving emergency therapies. The development of laboratory models that allow for the systematic screening and study of elements that contribute to BCBM is also required. Biomimetic strategies have been used to recapitulate brain microenvironments for studying BCBM [200]. In this paper, we will discuss invasion, intravasation, and extravasation, as well as the application of microfluidic techniques to studies pertaining to each of these stages.

The process of invasion starts when cancer cells begin to separate from the main mass of the tumor and enter the tissue that is around the tumor. Cancer cells eventually get restricted, hypoxic, and physiologically deprived as their growth rate continues to rise. These chemicals make cancer cells go through an epithelial-to-mesenchymal transition (EMT), which changes the shape of the cells and makes them move much faster [201, 202].

In an organ-on-a-chip system, which was developed to create a TME for the purpose of isolating the influence of pH on tumor viability, it was discovered that CaCO3 nanoparticles stimulated cancer cell reprogramming in order to suppress tumor growth and invasion. This was accomplished by inhibiting the spread of the tumor. CaCO3 nanoparticles were also used to treat BCCs (MDA-MB-231) that were cocultured with fibroblasts. The findings revealed that this treatment may inhibit the aggressiveness of tumor cells without influencing the development and activity of the stromal cells that are around the tumor [203]. Using a microfluidic technique for the assessment of the metastatic propensity of breast cancer samples, it was observed that high invasive capacity was connected with the RAS/MAPK and PI3K pathways [204]. Furthermore, it was noticed that cancer cells selectively invaded areas of a microfluidic device that had higher oxygen levels [205]. Through the use of microfluidic devices, it was found that tumor-associated macrophages and U-937 cells enhance invasion [206]. Additionally, it was discovered that breast cancer stromal cells communicate with migratory tumor cells to enable their motion by secreting MMPs at stages that are sufficient to overpower anti-MMP drugs [207].

In the direction of intravasation modeling, it has been shown that motile breast cells, after they are ready to migrate through the ECM, will track collagen fibers that link from their original location to neighboring blood or lymph vessels [208]. Nevertheless, since cadherins create tight intercellular junctions, BCCs are often unable to penetrate the basal lamina or the endothelial cell layer that surrounds the lumen of these vessels. This is one of the reasons why breast cancer is so difficult to treat [209, 210]. It appears that this may be circumvented with the aid of a variety of signaling pathways and interactions between macrophages, that together generate a micromilieu that makes it possible for cancer cells to enter the circulation. Macrophages in the surrounding environment of a tumor exhibit a diverse range of phenotypes, from those that are anti-tumor (M1) to those that are pro-invasion (M2) types [211]. Pro-invasion M2-like tumors release factors that decrease cadherin concentration in vascular endothelial cells. These factors include the angiogenic signals TNF1, VEGF, and EGF, as well as immune-cell recruiting signals such as the CXCL family. These factors all prime the vessel for permeation and are expressed by pro-invasion M2-like tumors [212]. The tumor cells themselves play an important role in the process of intravasation by using several mechanisms, including the NOTCH and TGF 1 pathways, to promote cadherin breakdown and endothelial contraction, which ultimately makes it possible for extravasation to occur [213]. Because of their involvement in promoting endothelial permeability, BCCs that secrete micro-RNA (miRNA) signals, such as miR-939, have been the subject of research [214].

As they provide accurate control over the spatial location of cells, microfluidic devices offer several advantages when it comes to the study of intravasation in breast cancer. MDA-MB-231 BCCs invasion via limited microchannels was demonstrated to elicit a shift in migration when tested in a constructed microfluidic migration chamber [215]. A microfluidic-based experiment was developed for the purpose of analyzing the control of carcinoma cell intravasation by biological factors from the communicating cells and cellular relations with macrophages. Endothelial permeability observations indicate that signaling with macrophages through the release of tumor necrosis factor-alpha (TNFα) results in endothelial permeability. Recreating the tumor–vascular interface in 3D enabled precise quantification of the endothelial barrier function [216]. In human-cell-based in vitro models, the 3D structural organization and the microenvironment of BCCs were reconstructed using a biomimetic microengineering method. In order to replicate the microarchitecture of breast ductal carcinoma in situ, the microsystem made it possible to co-culture breast tumor spheroids with human mammary ductal epithelial cells on one side of an ECM membrane and mammary fibroblasts on the other side in a compartmentalized microfluidic device [217]. The simultaneous investigation of BCCs invasion and intravasation as well as vasculature maturation influenced by tumor–vascular crosstalk led to the development of a 3D microfluidic platform consisting of concentric three-layer cell-laden hydrogels. This platform was created in order to facilitate research. It was established that the existence of a vasculature that had spontaneously generated contributed to an increased level of MDA-MB-231 invasion into the 3D stroma. Cancer cells that invaded the vessel drastically reduced its diameter while simultaneously increasing its permeability, and the primary signaling cytokines involved in tumor–vascular crosstalk that governs cancer cell invasion and intravasation were identified [218]. In order to dynamically observe the progression of the tumor, including cell migration, angiogenesis, and intravasation of tumor cells, a microfluidic platform that simulates biological mass transport near the arterial end of a capillary in the TME was developed. This allowed for dynamic observation of the progression of the tumor [219]. However, the majority of in vitro metastasis models emphasize examining blood-vessel-based metastatic routes. As a result, our knowledge of lymphatic metastasis, which is likewise strongly tied to the inflammatory system, is restricted. In this regard, a three-channel microfluidic device was created to simulate the lymph vessel–tissue–blood vessel configuration in order to get a better understanding of the impact that inflammatory cytokines have on lymphatic metastasis. Human umbilical vein endothelial cells and human lymphatic endothelial cells were seeded in the side channels to rebuild blood and lymph vessels, respectively. Interleukin 6 (IL-6) is an inflammatory cytokine that, when given to several subtypes of BCCs, triggers EMT and accelerates tissue invasion. With lymph vessel–tissue–blood vessel chips it should be possible to study how cells in the TME talk to each other in response to different outside factors like inflammatory cytokines, stromal responses, hypoxia, and lack of food [220].

Extravasation is the process by which CTCs leave the circulatory system to settle in a new organ system; it has been discovered that breast CTCs primarily extravasate into four organs, namely the bone, the brain, the liver, and the lungs, where they then form secondary tumors that can be fatal [221]. The scientific knowledge of CTC behavior, organotropism, and extravasation might be significantly advanced with the use of microfluidic devices, which could provide a potent tool. A microfluidic approach was developed for the integrated capture, separation, and analysis of membrane markers as well as for the quantification of proteins produced by single CTCs. It was discovered that the measured secretion level of granulocyte growth-stimulating factor (G-CSF), which plays a role in the recruitment of neutrophils, is substantially expressed across cancer cell types [222]. This technique was deployed in order to show the extravasation of CXCR4-expressing MDA-MB-231 cancer cells over a confluent HUVEC monolayer in the presence of a CXCL12 chemokine gradient. Control studies were provided to verify the fact that the migration of MDA-MB-231 cells was due to regulated chemotaxis rather than a random process. The way these experiments were described fit with the idea of organ-specific extravasation [223].

### BBB modeling in BCBM research

Unlike other organs, BCCs find it challenging to colonize the brain tissue as they have to overcome the BBB during extravasation. However, in many cases, aggressive breast cancers (especially TN and HER-2 positive breast cancers) are able to cross the BBB and colonize in the brain [224]. After extravasation through the BBB, BCCs interact with the brain ECM, which mainly comprises glycosaminoglycans, proteoglycans, and glycoproteins. Following brain metastasis, the median survival of patients drops asymptotically because anti-metastatic therapies cannot be effectively transported across the BBB. The BBB is made up of tightly packed brain microvascular endothelial cells that make up the vasculature. These cells are surrounded by pericytes, which are in contact with astrocytes in the brain parenchyma [225, 226].

Models that simulate the BBB’s well-organized and distinctive characteristics are of significant interest. It’s ideal for BBB models to have the same cell types and distribution as in vivo, to be able to express special proteins like enzymes, receptors, and transporters, to be able to simulate the mass transport mechanism and pathway [227].

The fact that in vivo models provide an experimental setting that accurately represents the intricacies of human physiology is the primary benefit associated with using these models. All of the studies take place in their natural setting and have the potential to yield a significant quantity of accurate statistics [228]. Nevertheless, there is no animal model that can accurately mimic all of the symptoms that are associated with human illness. As a result, these simulations can only be understood as a close approximation of human biology. One of the most significant drawbacks of using in vivo models is that it may be challenging to implement the findings that these systems provide to the world of humans. More than 80% of the findings gleaned from animal models do not, in a simple manner, match the reactions shown in humans [229]. Variability from animal to animal is also another source of concern. Furthermore, in order to track the entire mechanism by which a disease develops, it is necessary to use distinct animals at various phases of the process. This makes the studies costly owing to the expenditures on both labor and animals. Last but not least, while conducting studies on living subjects, researchers often make use of very high dosages of various drugs. These doses are too small to be used in high-throughput screening to find new drugs [227, 230].

On the other hand, in vitro cell culture models have been used for the purpose of conducting research on a variety of processes that support the physiology of the BBB for many years. Studies may be carried out in an environment that is precisely managed and regulated when cells such as endothelial cells, astrocytes, and pericytes are used. The technique of cell culture is straightforward, has a high rate of repeatability, and is well suited for high-throughput screening. The Transwell® systems are the most commonly used in in vitro models [231]. These models include the cultivation of one or more cell types on semi-permeable microporous inserts. From a functional point of view, the Transwell® versions are convenient and economical to use, making them a good choice. In order to make high-throughput drug permeability screening easier, these technologies may make it possible to reduce the number of animal studies needed, speed up tests, and use a less reagents and chemicals [232].

However, these systems fail to capture many aspects of in vivo physiology and the cellular milieu. To circumvent this issue, a cell-filled hydrogel mimicking the BBB model could be used to bioimitate the environment and function of the brain’s microenvironment. A hydrogel model permits cells of various sorts to come into contact with one another in a 3D architecture [149]. Work by Augustine et al. showed that a multilayered cell culture platform in metastasis models can be created by using methacrylated gelatin (GelMA) hydrogel with the mechanical support of Transwell® membranes, which can offer an ideal milieu for astrocytes and endothelial cells to grow. According to their findings, BCCs are capable of crossing the BBB model, nevertheless, treatment with cisplatin prevented these cells from spreading throughout the model [233].

Experiments investigating the BBB also make use of brain slice models. In these models, organotypic hippocampal slices are cultivated on a membrane surface, and the models are used to investigate how the BBB operates in response to a variety of normal and abnormal situations. Because brain slices include all cell types and interactions, these models offer full architectures that are useful instruments for the study of biological and pharmacological processes [234].

**Fig. 6:**
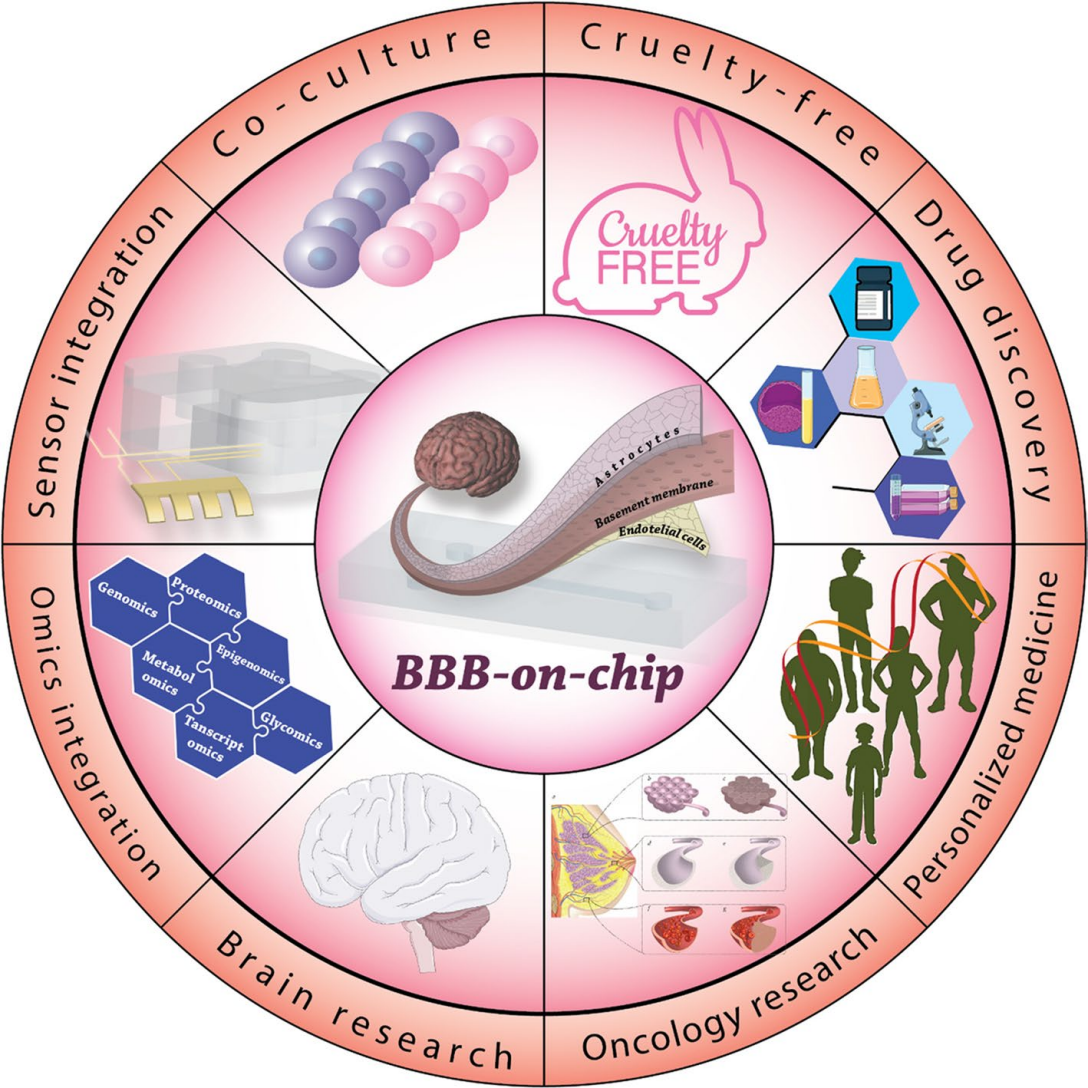
Applications and the technical advantages of BBB models. Devices based on microfluidics can imitate the human BBB’s complicated structure in vitro. These adaptable devices provide assistance for a broad variety of prospective applications, such as the creation of innovative treatments, personalized medicine, research in toxicity, and basic research on the brain. This method could also be a way to reduce the number of animals used in scientific research

Static models include Transwell® models, brain slice models, and other similar models that have no fluid flow. It is believed that endothelial cells are subjected to shear stresses when fluid flow is present, and these pressures are essential for correct endothelium polarization and the development of tight junctions. In this case, it is feasible to regulate the flow of fluid through microfluidic channels in a straightforward and precise manner by designing channels specifically based on the brain microenvironment [227]. Microfluidic BBB (µBBB) devices offer extensive possibilities for the regulation of the mass transport of signaling molecules, active agents, drugs, and nutrients, which are all relevant to the field of biological research (Fig. 6). In order to produce a microenvironment that is physiologically appropriate for the BBB, it is important to either minimize the amount of mass transport or optimize it. Researchers have found that the flow rate of the medium, the diameter of the microchannels, the porosity of the separation membrane, and the direction of flow all affect how mass moves in microfluidic platforms [235].

Both the pathology of brain tumors and the distribution of drugs across the BBB are thought to include a questionable functional component for the stability of the BBB. According to the findings of several studies, a failure in the integrity of the BBB facilitates the metastasis of cancer to the brain. Research that uses bioengineered µBBB systems, on the other hand, offers significance for the future development of therapeutic drugs that will lead to improved results for individuals with brain tumors. They also give information about this research and the development of systemic drugs that could be used to prevent brain metastases in people who are at risk [236, 237].

Interesting research defined a novel microfluidic model of the BTB and BBB that integrates components including flow and generated shear stress on the endothelial cells. The study used cells, including human umbilical vein endothelial cells co-cultured with CTX-TNA2 rat astrocytes (BBB model) or Met-1 metastatic murine BCCs (BTB model). With the use of a porous membrane, cells in microfluidic channels may interact with one another. This new microfluidic in vitro BTB model can recreate shear stress, permeability, and efflux activities in a way that is similar to how these things are studied in living organisms [238].

**Fig. 7:**
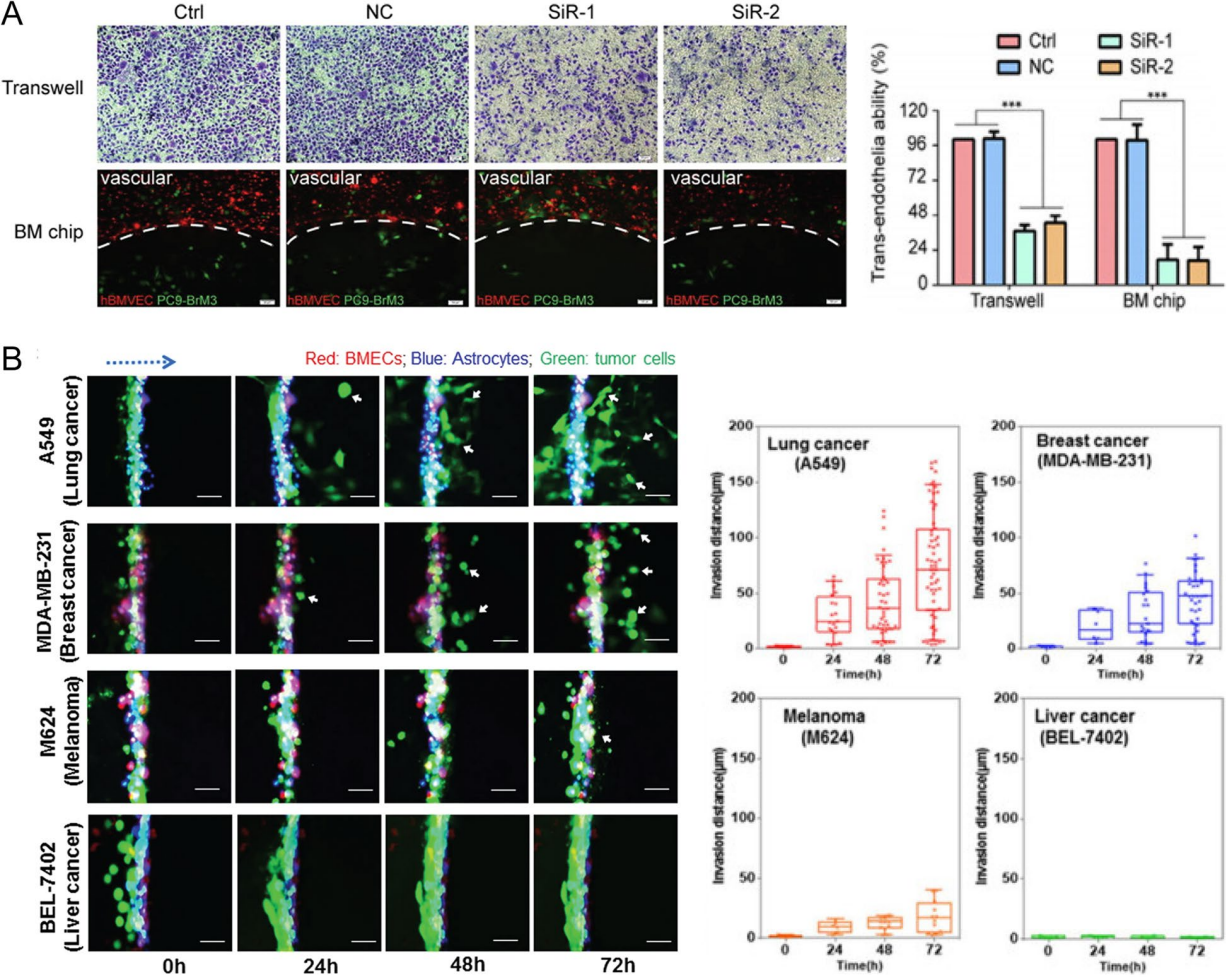
**A** Representative microscopic images demonstrating transendothelial migration on the Transwell® as well as BBB cell penetration on chip (upper and lower scale bar are 20 and 50 μm, respectively). **B** Time-lapse microscopic photos were taken over 72 h show the movement of a variety of cancer cells through the BBB system. Diagrams depicting the movement of different cancer cells pass through the BBB. Reprinted in part with permission from refs [239, 240]

In related research, investigators developed a dynamic 3D microfluidic system to replicate the characteristics of the human BBB (Fig. 7) [239, 240]. The components of the system have the potential to collaborate in order to replicate the properties of the BBB. That, in turn, paves the way for the examination of reactions in both normal and pathological microenvironments inside the brain [241]. Intercellular interactions, stimuli sensed by mechanoreceptors, and the mobility of individual cells are the means by which this goal may be accomplished. It has been shown that this technology is able to investigate brain metastases in human lung, breast, and melanoma cells, as well as those cells’ sensitivities to treatment. The findings showed that contact between cancer cells and astrocytes may inhibit the capacity of brain tumor cells to reach the circulatory system [241]. Non-endothelial neurovascular cells are very necessary components in the process of inducing BBB phenotypes and adjusting the dynamic responses of the BBB to brain activity. Many models of the µBBB would include neurovascular cell types in addition to brain microvascular endothelial cells. This would give researchers a wider range of options for studying the basic and complex molecular and cellular processes of BBB biology [235]. In the case of BCBM, some in vitro and in vivo studies were summarized in Tables 3 and 4, respectively.

**Table 3:** Some in vitro studies in the field of BCBM

Model	Polymer / cell	Outcome	Ref
BBB model	GelMA/ endothelial and astrocyte cell	The BCCs can migrate across the BBB model, Cisplatin (as a chemotherapy agent) prevented cancer cells migration across the BBB model, this model was a suitable tool for chemotherapy drugs screening	[233]
BBB model	brain-like endothelial cells and brain pericytes	The model has the high barrier properties, the integrity of the BBB is affected by serum of patients with cerebral metastases	[242]
	CD34 + cells differentiation from human umbilical cord blood and Pericyte/ Bovine Brain Capillary endothelial cells and Rat glial cells/ Mouse brain capillary endothelial cells and Mouse glial cells	Only model formed by human stem cells has BBB properties and measurable cell-interaction capacities	[243]
BBB and BTB brain metastases model	human umbilical vein endothelial cells, CTX-TNA2 rat brain astrocytes, and Met-1 metastatic HER2 + murine BCCs/ microfluidic chips	small and not efficacious amount of trastuzumab can cross the model, useful for detection and management of brain metastases	[244]
BCBM model	hyaluronic acid	brain metastatic BCCs has a dormant phenotype on soft hydrogel and proliferative phenotype on stiff hydrogel, stiffness-based dormancy was reversible	[149]
BCBM model	hyaluronic acid	Hydrogel stiffness has a direct effect on cancer cells proliferation, adhesion, and migration, focal adhesion kinase-phosphoinositide-3 kinase pathway mediated stiffness-based cell responses	[154]
BCBM model	PEG-maleimide	This model is useful for study the interactions between cancer cells and healthy cells, brain tissue stiffness has not a significant difference across species	[157]
BCBM model	patient-derived primary or brain metastasis cancer cells (organoids)	CAF promote cancer cells migration, blocking CXCL12 and CXCL16 is useful for preventing BCBM	[146]
BCBM model	MDA-361 cell line	MDA-361 spheroid has a faster growth rate compared to monolayer culture, tight, highly reproducible 3D spheroids	[134]

**Table 4:** Some in vivo studies in the field of BCBM

Model	Cell line/animal	Outcome	Ref
BCBM model	MA11/athymic nude mice	Brain metastasis occurred 65 days after injection of MA1 I in the left ventricle of mice. Serum MUC1 levels produced by MA1 I cells are associated with brain metastasis. This model is useful for investigating the preferential mechanisms for metastases. This modeling did not cause bone, liver, kidney, spleen, and heart metastases	[60]
BCBM model	MDA-MB-231BR or MDA-231P / nude mice	MDA-231BR was metastases exclusively in the brain and did not cause bone metastases MDA-231P metastasized to the brain, bone, adrenal glands, and ovaries Provide a useful model for identifying new genes or molecules responsible for metastasis	[61]
BCBM model	MDA-MB-231 BR1, -BR2 and -BR3 / nude mice	Increased VEGF-A is a feature of brain metastatic cells Mice injected with metastasis-selected cells had a shorter mean survival than mice injected with the main cell line The VEGF-receptor tyrosine kinase inhibitor reduces brain metastases	[64]
BCBM model	JIMT-1-BR3/ NRC nu/nu mice	Temozolomide significantly prevented brain metastasis	[67]
BBB and BCBM model	CN34-BrM2/ nude mice	EGFR and COX2 ligands are involved in brain and pulmonary metastases ST6GALNAC5 mediates brain metastasis exclusively, and its expression in BCCs increases their adhesion to brain endothelial cells and their passage through the BBB	[65]
BBB and BCBM model	SUM190-BR3/ Athymic NIH nu/nu mice	Targeting pericytes, Desmin, and α2 laminin are effective on BBB permeability and thus increase the effectiveness of chemotherapy	[80]
BCBM model	SKBrM3 + / nude mice	Cabozantinib and neratinib inhibited cell proliferation and migration; and inhibited tumor growth and brain metastasis This model is a valuable tool for drug screening of brain metastases	[83]

## Conclusion and future perspectives

Patients who have breast cancer often may face metastatic condition that has settled in the CNS. Different treatment techniques for brain metastases may need to be developed due to the BBB structure and the particular microenvironment of the brain. In vivo models offer a dynamic environment that includes the immune system, vasculature, and other naturally occurring processes in the tumor microenvironment; nevertheless, the preparation process for these models is time-intensive, difficult, and expensive. In vitro models are more straightforward, making it feasible to analyze results more easily and investigate the possible factors in greater depth. Using engineered 3D models, mostly hydrogels, and numerous cell types at the same time in the form of co-cultures, these cells can adapt their natural morphology and accomplish cell–cell and cell-ECM interactions. Microfluidics is a useful solution for the construction of 3D in vitro models. This system offers spatial control over the biochemical composition, cells, and fluid flow, making it one of the most promising manufacturing methods. This method has the benefit of being able to create 3D models that are very similar to the microenvironment of metastatic brain tumors.

## Data Availability

All the data and materials supporting the conclusions were included in the main paper.

## Abbreviations

BCBM: Breast cancer brain metastasis
ECM: Extracellular matrix
2D: Two-dimensional
3D: Three-dimensional
IDCs: Invasive ductal carcinomas
ILCs: Invasive lobular carcinomas
CNS: Central nervous system
IHC: Immunohistochemical staining
EGFR: Epidermal growth factor receptor
HER2: Human EGFR2
CK5/6: Cytokeratin 5/6
PR: Progesterone receptors
ER: Estrogen receptors
TN: Triple-negative phenotype
BCCs: Breast cancer cells
HRG: Heregulin
HPSE: Heparanase
EpCAM: Epithelial Cell Adhesion Molecule
CTCs: Circulating tumor cells
BTB: Blood-tumor barrier
BMCLs: Brain metastatic cell lines
BRCA1: Breast cancer gene 1
PTEN: Phosphatase and tensin homolog
CDK: Cyclin-dependent kinase
SMAD4: SMAD family member 4
VEGF-A: Vascular endothelial growth factor A
PDX: Patient-derived xenografts
CAFs: Cancer-associated fibroblasts
HA: Hyaluronic acid
PCL: Poly(ε-caprolactone)
PLGA: Poly(lactide-co-glycolide acid)
PDMS: Polydimethylsiloxane
PMMA: Poly(methyl methacrylate)
PS: Polystyrene
PC: Polycarbonate
iPSCs: Induced pluripotent stem cells
EMT: Epithelial-to-mesenchymal transition
TNFα: Tumor necrosis factor-alpha
IL-6: Interleukin 6
GelMA: Methacrylated gelatin
µBBB: Microfluidic BBB
MAPK: Mitogen-activated protein kinase
MMP-2: Matrix metalloproteinase-2
cGMP: Cyclic guanosine monophosphate adenosine monophosphate
INFα: Interferon alpha
STAT1: Signal transducer and activator of transcription 1
NF-κB: Nuclear factor kappa-light-chain enhancer of activated B cells
PI3K: Phosphatidylinositol 3-kinase
RB1: Retinoblastoma protein 1
TME: Tumor microenvironment
